# Relationship between workplace violence and work stress in the emergency department

**DOI:** 10.5249/jivr.vo112i2.1526

**Published:** 2020-07

**Authors:** Lahya Afshari Saleh, Shabnam Niroumand, Zohreh Dehghani, Tahoura Afshari Saleh, Seyyed Mohammad Mousavi, Hosein Zakeri

**Affiliations:** ^*a*^ Occupational Medicine Department, Division of Sleep Medicine, Psychiatry and Behavioral Sciences Research Center, Mashhad University of Medical Sciences, Mashhad, Iran.; ^*b*^ Department of Community and Family Medicine, Faculty of Medicine, Mashhad University of Medical Sciences, Mashhad, Iran.; ^*c*^ Department of Occupational Medicine, Faculty of Medicine, Mashhad University of Medical Sciences, Mashhad, Iran.; ^*d*^ Sabzevar University of Medical Sciences, Sabzevar, Iran.; ^*e*^ Department of Emergency Medicine, Faculty of Medicine, Mashhad University of Medical Sciences, Mashhad, Iran.

**Keywords:** Workplace violence, Occupational stress, Emergency department staff

## Abstract

**Background:**

Work place violence (WPV) is one of the workplace factors that can affect many aspects of the emergency staff's life. In this study, we are investigating the relationship between WPV and occupational stress.

**Methods:**

We surveyed emergency department (ED) staff in a cross-sectional study design in three Mashhad Hospitals between 2017 and 2018. World Health Organization WPV and occupational stress questionnaire was used and 171 out of 200 collected data were analyzed. To investigate the relationship between different variables, t-test and logistic regression were implemented.

**Results:**

In general, 58 (34.5%) participants had reported a physical assault, 116 (71.6%) verbal abuse, and 76 (44.4%) bullying/harassment within the past year. Males reported more experience of physical assault (P less than 0.001), verbal abuse (P less than 0.04) and bullying/harassment (P less than 0.01). The educational level and time shifts were associated with the frequency of physical violence and bullying/harassment (P less than 0.03), respectively. We noticed an association between the job stress scales and some types of work place violence including physical assault (P=0.02), bullying/harassment (P=0.006) and demands scale in recent cited violence (P=0.07).

**Conclusions:**

We presented considerable prevalence of WPV among ED staff. Improving workplace condition and reducing occupational stress could be decrease WPV frequency.

## Introduction

One of the most demanding subjects that healthcare organizations globally facing is work place violence (WPV).^1^ WHO defines WPV as “Incidents where employees are abused, threatened, assaulted or subjected to other offensive behavior in circumstances related to their work”.^2^ Previous studies indicating that 25 percent of WPV occurs in the healthcare sectors and more than fifty percent of workers in these section have experienced at least one episodes of work violence (either physical or psychological) during their career.^3,4^ Like Canadian Nursing Advisory Committee health care workers, that were prone to experiencing work violence even more than law enforcement.^5^

Different studies indicated the prevalence of different types of WPV, especially verbal among nurses^6,7^ with reported rates of 27% in Iran,^8^ 89.6% in Egypt, 85.2% in Turkey, 75.8% in Bulgaria, 67.2% in Australia, 61% in Australia, 54% in Thailand, and 61% in South Africa.^9^ A review of 136 articles provided data on 151,347 nurses from 160 samples. This review indicated an incident rate of 36.4% for physical violence, 66.9% for nonphysical violence, 39.7% for bullying, and 25% for sexual harassment. Interestingly, the exposure rates fluctuate geographically. The highest rates of nonphysical violence and bullying reported in the Middle East, meanwhile, the highest rates for physical violence and sexual harassment were occurred in the Anglo region.^10^ WPV mostly happened in waiting rooms, geriatric units, psychiatric wards and emergency departments (EDs).^11^ Among all of these sections, EDs, which is characterized by their 24-hour accessibility, limited security, and highly stressful environment, own the largest share of work violence. ^12^

Contrary to studies, which provide ample evidence for serious and destructive consequences of WPV toward medical health workers. We are witnessing inadequate educational programs, security guards, suitable policy and laws, and an efficient reporting system to prevent the incidence of violence toward Iranian medical staff. Recently a study in Iran showed that more than a quarter of nurses respond to WPV by “taking no action” and the small minority of them (0.5%) consider suing the violent person.^13^ This shows that a safe workplace environment is crucial for enhancing performance and cost reduction in the mental health sector.^14^ The safe environment comes with directing attention and resources by discussing safety, offering safety training and having the safety policies. ^15^

Among the different parts in hospitals, the highest incidence of WPV occurs in emergency departments (EDs). The EDs is documented as an area at special risk of violence due to twenty four hours accessibility, lack of adequately trained, armed, or visible security guards and a highly stressful work area. The overwhelming majority of perpetrators of EDs violence are patients, their family members and visitors. The unanticipated nature of illness such as acute illness and trauma, patient pain and discomfort, as well as the tension, stress, and anger of patients and their relatives, and adverse unexpected outcomes such as death are often compounded by cramped space, lack of privacy and intense interpersonal interactions, and long waiting times for consultation or admission. 

A recent review on 68 studies with more than 100 thousand participants suggested that the most profound consequences of WPV were psychological emotions (including symptoms of posttraumatic stress disorder, depression, anxiety) and the negative emotions (like anger, fear, and sadness). Reviewing literature informs us about the profound consequences which WPV has on nurses, that might last month and even years after its occurrence.^16,17^ Research on EDs personnel found a 12% rate of clinical post traumatic stress disorder (PTSD) which is significantly above the general population.^18,19^

Occupational stress is a result of the mismatch between an individual and the environment as the harmful physical and emotional responses that occur when the requirements of the job do not match the capabilities, resources, or needs of the worker. Generally, higher discrepancy between external stresses and an individual’s capabilities caused experiencing the higher the level of stress. On the other hand, work stress is linked with physical and mental health risks.^1,2^ Work stress attitudes a financial load to organizations and society at large, caused by productivity loss due to sickness absence, work disability benefits, and health care costs.^4^ Nursing is known to be a very stressful occupation throughout the world under great pressure due to heavy workload, contending with death and dying, inter-staff conflict, lack of resources, and insufficient organizations.^1-3^ One of the most common experiences among health care workers in EDs as a result of aggressive behavior is stress symptoms which can undermine job satisfaction and performance.^20-27^ On the other hand increased occupational stress might be a predisposing factor for WPV. High job demand, low job control, conflict with coworkers, shift work, low social support as the occupational stress components can facilitate WPV occurrence. The more negative stress is generated the greater violence.^2,28,29,30^ Also different kinds of emotions (like anger and embarrassment, etc.) experienced by the nurses as a result of the aggressive incidents can influence their behavior and beget more WPV. ^17^

Another subject that has been investigated in the field was the role of gender in WPV. Some studies in this field suggested more experience of physical assaults for men than women, while more women reported verbal threats in inpatient settings.^31^ Meanwhile, another reported similar pattern of violence across genders.^32^

Although data support the relationship between stress and WPV, it is not clear which kind of work violence is more associated with the work related stress and how occupational stress and work environment affect the frequency of WPV. So, this study aimed to investigate the relationship between WPV and work related stress in three biggest hospitals in Mashhad city as the biggest part of emergency departments in Northeast of Iran. 

## Methods 

**Study participants**

This analytical cross-sectional study carried out in three educational hospital centers in Mashhad (the second most populated city of Iran and the capital of Razavi Khorasan province, located in the Northeast of Iran) between October 2017 and February 2018. Each hospital placed in a different geographical position and socio-economic context. The study participants were emergency department staffs and data were collected through two different questionnaires. The items were prepared in the form of multiple choices and short answer questions. The self-reporting questionnaires were filled out with approval forms handed by the blinded supervisor from different hospital in emergency department and participants were asked to give the questionnaires back to the second supervisor that managed completing the forms by staffs solely. Both supervisions were blinded about process of research, anonymity of study participants and analyzing documents. 215 questionnaires were administrated in emergency department. Among 200 questionnaires that were collected, 171 have been analyzed, the rest were filled out incompletely. So the response rate in this study was 79%.

**Data gathering**

The study questionnaire contained two parts:

1. World Health Organization WPV questionnaire comprises 78 questions. The first part of the questionnaire contains questions about personal and workplace features. The second part asks about experiencing any physical violence, in the past 12 months which is defined as any intentional act that causes physical damage to another person. And the last part contains questions about psychological violence (verbal violence, bullying, and any type of harassment) experience within last year. This questionnaire is translated and validated to Persian.^33^

2. The HSE (The Health and Safety Executive) occupational stress questionnaire: the HSE's scale includes 35 questions with 7 subscales which designed by the England health and safety organization to assess occupational stress of workers and employees.^34^ Thirty five questions in this tool are fit in 7 domains, including demand (8 items), control (6 items), managerial support (5 items), peer support (4 questions items), relationships (4 items), role (5 items) and change (3 items). The items of the HSE questionnaire include a 5-point Likert Scale (never, rarely, sometimes, often and always). The score of each question ranged from 1 to 5, under which 1 indicates undesirable, and 5 refers to desirable state.^35-37^ This questionnaire was translated and validated in Iran.^36,37^ Moreover, they have reported Cronbach's alpha of 0.78 for this scale. Besides, Akbari et al. reported the interclass correlations for the domains ranged from 0.52 to 0.73 with a median of 0.7. The confirmatory factor analysis also showed that the Persian version of the HSE questionnaire had adequate construct validity.^35^

**Statistical analysis**

To analyze the data independent samples t-test were implemented. Differences between demographic characteristics also were investigated. Data were analyzed using IBM SPSS Version 16 Software. A multiple logistic regression analyses were conducted to examine the effective factors related to experiencing work place violence. All variables with P value <=0.5 in univariate analysis were entered in model. These variables include different domain of HSE questionnaire except manager and peers support, role and changes, sex, hospital, time sheet and age. Finally, logistic regression model and stepwise selection was used to evaluate the significant independent variables

## Results

The mean age of the study participants was 31.62 years (age range, 20-50 years). There were 89 males (52%) and 82 females (48%) with average work experience of 6.8±5.17 years. Average years of experience among employment were divided under three categories. Demographic characteristics of study participants presented in Table 1.

**Table 1:** Demographic characteristics of study participants.

Variables	Numbers (%)	Variables	Numbers (%)
**Gender**		**Profession**	
Male	89 (52)	Nurse	62 (36.3)
Female	82 (48)	Resident	27 (15.8)
		Intern	17 (9.9)
		Nurse aid	61 (35.7)
		Guard	4 (2.3)
**Marital status**		**Time sheet**	
Single	48 (28.2)	Day work	30 (17.5)
Married	122 (71.8)	Rotating work	141 (82.5)
**Education**		**Age Category (year)**	
Under bachelor	129 (74.43)	20->=30	81 (47.6)
Master's degree and upper	42 (24.56)	30->=40	79 (46.5)
		40->=50	10 (5.9)
**Hospital**		**Employ Category (year)**	
A	70 (40.9)	1->=10	139 (81.3)
B	35 (20.5)	10->=20	29 (17)
C	66 (38.6)	20->=30	3 (1.8)

The features of the participants based on the different types of experienced violence were listed in Table 2. In general, 34.5% (58) of participants had reported physical assault, 71.6% (116) verbal abuse and 44.4% (76) bullying/harassment. As shown in Table 2, gender differences were reported within all forms of work-violence. Particularly, males reported more experience of physical assault (P<0.001), verbal abuse (P<0.04) and bullying/harassment (P<0.01). As Table 2 indicating marital status was not significantly related to the experience of work-violence; along the same line, profession, age, and work experience were not differentiated in the experience of work-violence. Educational level was differentiating statistically significant only in physical abuse (P<0.005), but not in verbal abuse or bully. Chart indicating that workplace circumstances also, had affected employee's experience of WPV. Differences in time shifts also show that working on rotational shift increase the chance of experiencing work-violence.

**Table 2:** Relationship between WPV and demographic characteristics

Variable		Physical assault N(%)	p	Verbal abuse N(%)	p	Bullying/ harassment N(%)	p
Gender	Male	42(72.4)	<0.001	68(58.6%)	0.04	48(63.2%)	0.01
	female	16(27.6)		48(41.4%)		28(36.8%)	
Marital status	Single	15(25.9)	0.54	29(25%)	0.1	19(25%)	0.55
	Married	43(74.1)		87(75%)		58(75%)	
Education	Under bachelor	24(41.4)	0.005	31(26.7%)	0.93	22(28.9%)	0.78
	Master's degree and upper	34(58.6)		85(73.3%)		54(71.1%)	
Hospital	A	36(62.1)	<0.001	58(50%)	0.01	43(56.6%)	0.001
	B	9(15.5)		24(20.7%)		15(19.7%)	
	C	13(22.4)		34(29.3%)		18(23.7%)	
Job title	Nurse	27(37.9)	0.1	42(36.2%)	0.7	30(39.5%)	0.57
	Resident	7(12.1)		16(13.8%)		11(14.5%)	
	Intern	2(3.04)		14(12.1%)		4(5.3%)	
	Nurse aid	25(43.1)		42(36.2%)		30(39.5%)	
	guard	2(3.4)		2(1.7%)		1(1.3%)	
Work shift	Day work	6(10.3)	0.08	18(15.5%)	0.77	7(9.2%)	0.03
	Rotating work		52(89.7)		98(84.5%)		69(90.8%)	
Age category	20_30	29(50.9)	0.56	54(47%)	0.82	34(45.3%)	0.81
	30_40	26(45.6)		53(46.1%)		36(48%)	
	40_50	2(3.5)		8(7%)		5(6.7%)	
Working Experience	1_10	44(75.9)	0.4	94(81%)	0.54	61(80.3%)	0.93
	10_20	13(22.4)		19(16.4%)		13(17.1%)	
	20_30	1(1.7)		3(2.6%)		2(2.6%)	

Averages scores of seven HSE scales in participants who had experienced a different form of violence were reported in Table 3. In all scales, attendants who mentioned having a history of violence had a lower score than the others but these differences were not statistically significant except for relationship scale in physical assault (P=0.02) and in bullying/harassment (P=0.006) and also for demands scale in recent cited violence (P=0.07). 

**Table 3:** Average scores of different HSE scales in participants with or without WPV.

	Physical assault			Verbally abuse			Bullying/harassment		
	Yes Mean(SD)	No Mean(SD)	P	Yes Mean(SD)	No Mean(SD)	P	Yes Mean(SD)	No Mean(SD)	P
**Demands**	6.18(1.51)	6.31(1.40)	0.58	6.28(1.60)	6.16(1.03)	0.65	6.04(1.38)	6.43(1.45)	0.07
**Control**	9.89(4.44)	10.35(4.2)	0.51	10.06(4.18)	10.34(4.55)	0.71	9.54(4.35)	10.59(4.26)	0.12
**Managers’ support**	9.07(4.75)	9.86(5.18)	0.33	9.13(4.77)	10.03(5.44)	0.30	9.03(4.79)	9.98(5.21)	0.22
**Peer support**	11.44(5.32)	11.66(5.28)	0.79	11.40(5.11)	11.47(5.50)	0.92	11.68(5.21)	11.52(5.30)	0.84
**Relationships**	6.48 (1.21)	7.40(3.00)	0.02*	6.87(2.23)	7.60(3.34)	0.1	6.48(1.23)	7.55(3.17)	0.006*
**Role**	14.89(5.12)	15.11(4.63)	0.78	15.04(4.79)	15.12(4.85)	0.92	14.75(4.81)	15.44(4.72)	0.35
**Change**	7.95(5.33)	8.59(5.10)	0.44	8.22(5.19)	8.50(4.95)	0.76	7.85(5.19)	8.70(5.14)	0.29
**Whole scale**	9.39(2.65)	9.90(2.51)	0.24	9.54(2.42)	9.93(2.72)	0.39	9.39(2.55)	9.98(2.54)	0.14

* P value less than 0.05 considered as significant level

Results of logistic regression in Table 4 indicated that the probability of experiencing violence for males was 3.06 (1.26-7.45) times more than females (P=0.01). This probability also increases by the workplace environment; as participants in hospital C had 3.64 (1.34-9.86) times (P=0.01) more probability for experiencing job violence compared with hospital A. 

**Table 4:** Demographics and workplace factors significantly associated with WPV.

Variable	OR	CI	P
Age	0.89	0.81-0.99	0.03*
Male	3.06	1.26-7.45	0.01
Hospital B	0.78	0.21-2.81	0.7
Hospital C	3.64	1.34-9.86	0.01*

Reference groups: female / hospital A *significant level considered as P value less than 0.05

## Discussion

The study aimed to investigate the frequency and correlations of WPV, which is one of the most prevalent incidents in the healthcare system, and work stress in emergency department of hospital. Recently, EU-OSHA (European Agency for Safety and Health at Work) reported psychosocial risks besides harassment and violence in the healthcare sector.^38^ In this study, our results showed that 34.5%, 71.6% and 44.4% of study participant in EDs had reported a physical assault, verbal abuse and bullying/harassment within the past year, respectively. Also, our findings showed that there was link between work stress scales and workplace violence in all scales and participants with history of violence had none significantly lower score than the others. 

In comparison to previous studies ^36-39^ on high prevalence of WPV in this study, we can explain this rate due to the low control and high physical demand over tasks, risky and time-consuming jobs among workers' in our investigated emergency department.

Previous investigations found that the association between WPV and occupational stress seems independent of marital status, profession, age and, work experience and our findings were in good agreement with previous investigations.^25^ According to our findings, gender, time shifts, and work place setting seems to play a crucial role in experiencing WPV. 

As previously mentioned, stress can aggravate violence by making staff more vulnerable to violent cues. Specifically, the experience of stress will cause less tolerance and more Ego depletion,^39^ which increases the proneness to violence;^40^ this will cause a closed loop of aggression. This is in line with our results, as man reported more experience of violence than woman (our findings showed that males reported more WPV than females). Since the previous studies had reported males used fewer coping strategies including both emotional focus and problem focus strategies under stress.^41^ This proposition is also align with Whittington and Wykes cyclical model of WPV of nurses.^42^ This model indicates that stress caused by exposure to violence head to impaired staff performance and adoption of manners which make the recurrence of violence more likely. Moreover, in some city areas, WPV is more acceptable toward men than women. Thus, we should expect intervention based on the patient's cultural background.

In our study, one of the key findings was the differences in WPV and occupational stress according to the workplace environment. This finding also is compatible with previous studies emphasizing the crucial role of the work environment of mental health workers in stress perception and violent incidents.^14,15^ One might suggest that these differences between hospitals A and C were because of differences in the socio-economic status of patients referring to these hospitals. So, we suggest that further studies in hospitals A and C would clarify possible risk factors and stressors that can be illuminating for policymakers to develop better prevention strategies for decreasing WPV and enhancing job satisfaction. This idea becomes more important when our data show that within different subscales of the HSE, the relationship is one of the important predictors of experience of physical assault and being bullied and harassed. Managing the relationships at work, include promoting positive working practices to avoid conflict and dealing with unacceptable behavior, can be learned and enhanced during different practices.^42-44^

Another factor that should be accounted for WPV is sleep deprivation. Night work and long shifts lead to more decrease of self-control and tolerance while increasing hostility and end up in increased WPV.^45^ Our results confirm this process by indication of significant differences in bullying/harassment within different time shifts. 

About searching HSE scale on study participants, we found that among different HSE scale just “relationships” factor was statistically significantly associated with WPV characteristics. This factor was lower than the other HSE scale among participants with positive WPV. The findings of regression modeling showed that “relationship” was one of the negatively predicting factors of WPV. This variable is related to establishing changes in the work place and workers ' knowledge. Misinterpretation of the quality of this factor “relationships” and their subsequent effects on the job and workplace could be main to stress among workers into different places.^46^ The results of previous studies showed that the worker is improvised for changes (about their work environment or work resources) and they do not given enough knowledge before applying these changes can cause occupational stress.^34,47^

We concluded the profound relationship between WPV and work place stress within emergency departments. Based to previous investigations, our study results were in agreement with previous ones and most of the participants had reported some forms of WPV in EDs. But one thing that needs more investigation is the relationship between WPV and occupational stress while some scholars^24^ suggested that occupational stress is the aftermath of WPV, others proposed that stress in the workplace environment triggers aggression. So, it is important to be careful in the interpretation of these correlational data. Differences between the frequency of WPV in the health care system and the differences in the incident of violence in different workplace settings suggest that this might be to some extent due to the different environmental conditions. So, we are suggesting that paying attention to specific environmental stressors besides personal abilities for interacting with these factors can be fruitful for policymakers.

Our study had several limitations that need to be mentioned for further studies. Our study limited with the small sample size generally according to work status and workplace. However, we selected the mentioned hospitals and participants randomly but we are proposing for future studies a larger sample size randomly related to selected hospital and work status. Another limitation of our study was the survey research design that was implemented. We suggest an experimental design for future studies to tackle the issue more precisely. Our study indicated that healthcare worker's coping skills probably playing a crucial role in WPV; thus, we propose future studies focusing on the role of these skills and WPV and environmental stressors. 
